# Metabolic Network Models of the *Gardnerella* Pangenome Identify Key Interactions with the Vaginal Environment

**DOI:** 10.1128/msystems.00689-22

**Published:** 2022-12-13

**Authors:** Lillian R. Dillard, Emma M. Glass, Amanda L. Lewis, Krystal Thomas-White, Jason A. Papin

**Affiliations:** a Department of Biochemistry and Molecular Genetics, University of Virginia, Charlottesville, Virginia, USA; b Department of Biomedical Engineering, University of Virginia, Charlottesville, Virginia, USA; c Department of Obstetrics and Gynecology, University of California—San Diego, La Jolla, California, USA; d Evvy, New York, New York, USA; University of Massachusetts Medical School

**Keywords:** *Gardnerella*, bacterial vaginosis, metabolic modeling, women’s health

## Abstract

*Gardnerella* is the primary pathogenic bacterial genus present in the polymicrobial condition known as bacterial vaginosis (BV). Despite BV’s high prevalence and associated chronic and acute women’s health impacts, the *Gardnerella* pangenome is largely uncharacterized at both the genetic and functional metabolic levels. Here, we used genome-scale metabolic models to characterize *in silico* the *Gardnerella* pangenome metabolic content. We also assessed the metabolic functional capacity in a BV-positive cervicovaginal fluid context. The metabolic capacity varied widely across the pangenome, with 38.15% of all reactions being core to the genus, compared to 49.60% of reactions identified as being unique to a smaller subset of species. We identified 57 essential genes across the pangenome via *in silico* gene essentiality screens within two simulated vaginal metabolic environments. Four genes, *gpsA*, *fas*, *suhB*, and *psd*, were identified as core essential genes critical for the metabolic function of all analyzed bacterial species of the *Gardnerella* genus. Further understanding these core essential metabolic functions could inform novel therapeutic strategies to treat BV. Machine learning applied to simulated metabolic network flux distributions showed limited clustering based on the sample isolation source, which further supports the presence of extensive core metabolic functionality across this genus. These data represent the first metabolic modeling of the *Gardnerella* pangenome and illustrate strain-specific interactions with the vaginal metabolic environment across the pangenome.

**IMPORTANCE** Bacterial vaginosis (BV) is the most common vaginal infection among reproductive-age women. Despite its prevalence and associated chronic and acute women’s health impacts, the diverse bacteria involved in BV infection remain poorly characterized. *Gardnerella* is the genus of bacteria most commonly and most abundantly represented during BV. In this paper, we use metabolic models, which are a computational representation of the possible functional metabolism of an organism, to investigate metabolic conservation, gene essentiality, and pathway utilization across 110 *Gardnerella* strains. These models allow us to investigate *in silico* how strains may differ with respect to their metabolic interactions with the vaginal-host environment.

## INTRODUCTION

Bacterial vaginosis (BV) is one of the most common vaginal conditions in reproductive-age women with vaginal complaints (1). BV is a polymicrobial condition of the vagina characterized by low levels of *Lactobacillus*, high levels of diverse anaerobes, a vaginal pH of >4.5, thin vaginal discharge, and a fishy odor (2). In North America, BV disproportionately impacts women of color. It is prevalent among black (33 to 64%) and Hispanic (31 to 32%) women compared to their white counterparts (23 to 35%) (3–7). The estimated annual health care-associated costs for BV treatment globally are $4.8 billion and an additional $9.6 billion when accounting for BV-associated HIV infection and BV-associated preterm birth (4). The bacterial etiology of BV and the mechanisms of pathogenic outcomes remain largely ill defined. However, since its literature debut in the 1950s as “Haemophilus vaginalis,” *Gardnerella* has consistently been reported as being one of the dominant genera in the vagina during BV (8).

The healthy vagina exhibits normal epithelial and mucosal turnover as a protective mechanism that can help eliminate unwanted colonizers (9, 10). A vaginal microbiome dominated by *Lactobacillus* is associated with healthy outcomes. This association of *Lactobacillus* with healthy outcomes is often attributed to lactic acid production creating an acidic environment inhospitable to many microorganisms (11, 12). Sexual activity, menstruation, hygienic behaviors, hormone fluctuation, antibiotics, and douching can cause changes in the vaginal microbiome and potentially open new niches for pathogens (13–15). Despite BV’s pervasiveness and its impact on women’s health, treatment options are limited and often ineffective. Typical antibiotic courses, specifically metronidazole and clindamycin, present initial success in clearing infection; however, 50% of women experience BV recurrence within 12 months of treatment cessation (16). The ineffectiveness of current treatment options highlights the need for deeper insight into the function of BV-associated organisms such as *Gardnerella*.

There is a significant lack of understanding of the functional metabolism of *Gardnerella*, a bacterial genus that often dominates the vaginal niche in BV. The *Gardnerella* pangenome remains largely uncharacterized, as noted by the rapidly evolving species classifications within this genus (17). Metabolic predictions using *in silico* analysis offer a unique opportunity to study taxonomic relatedness based on inferred function as opposed to the traditional genetic content-based approach. Previous research has shown that women who are positive for BV typically present with multiple strains of *Gardnerella* (18, 19). While multiple, as well as different, strains are present during BV, how these strains differentially interact with the vaginal metabolic environment and the secondary implications for pathogenesis are not well characterized. By understanding these strain-level differences, we can begin to define the driving metabolic factors of strain-level cocolonization and predict how they interact in BV.

**FIG 1:**
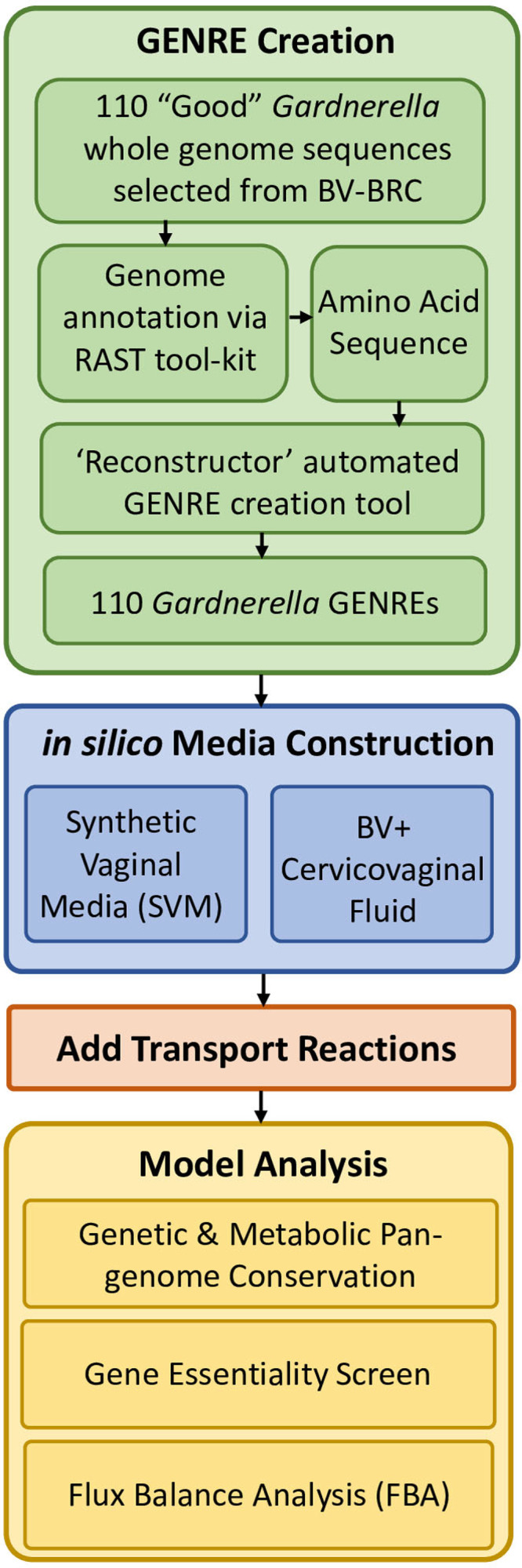
Analysis workflow for pangenome model network reconstruction, contextualization, and analysis.

Here, we present the first characterization of *in silico* models reflecting the genetic content and metabolism of the *Gardnerella* pangenome using genome-scale metabolic network reconstructions (GENREs) to allow the identification of potential antibiotic targets, both strain specific and conserved, and to make predictions regarding differential pathogenesis (Fig. 1). By defining the conserved metabolic functions and strain-level variation within the *Gardnerella* pangenome, we can begin to make testable predictions about microbial physiology and give structure to the heterogeneous nature of BV.

## RESULTS

### Strain comparisons.

**FIG 2:**
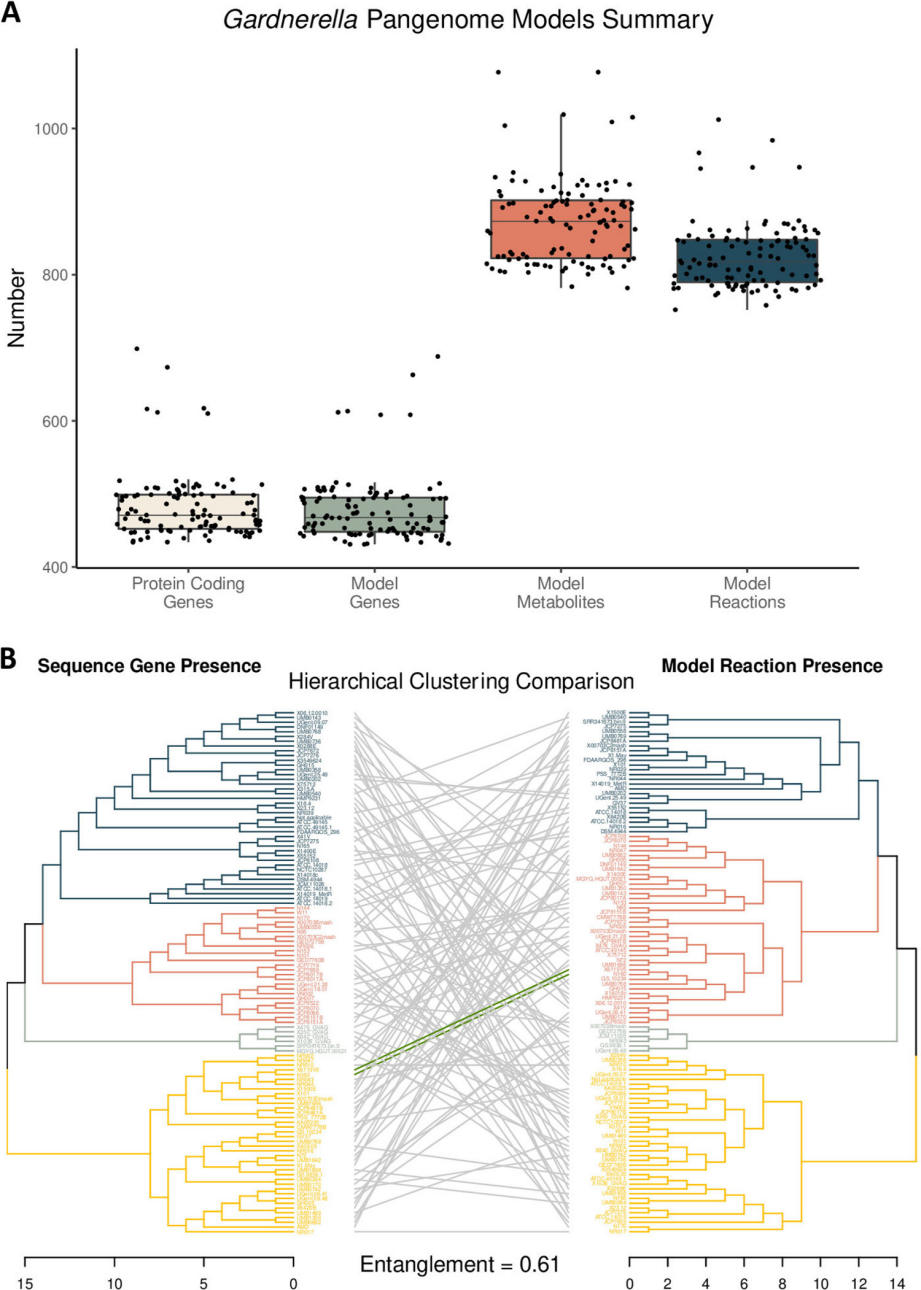
(A) Summary of the pangenome sequence content and the corresponding metabolic model content. (B) Comparison of hierarchical clustering based on protein-encoding gene presence (left) and model reaction presence (right). The branch color indicates the associated k-means group. Green entanglement lines indicate that the strain was placed in the same branch for both dendrograms (note that there are only 2 strains that cluster into the same groups).

The number of protein-encoding genes across the 110 *Gardnerella* strains ranged from 434 to 1,012, with a median value of 471. The number of genes in the 110 metabolic models ranged from 431 to 688, with a median value of 468. The number of model metabolites ranged from 782 to 1,077, with a median value of 873. Finally, the number of model reactions ranged from 752 to 1,012, with a median value of 818. As shown in Fig. 2A, there are consistently 6 outlier strains across all four categories. A comparison of the hierarchical clustering of strains based on the full genetic content (including core and peripheral genes) versus the full metabolic content results in an entanglement value of 0.61, which indicates 61% dissimilarity between the two dendrograms (Fig. 2B).

### Genetic and reaction conservation.

**FIG 3:**
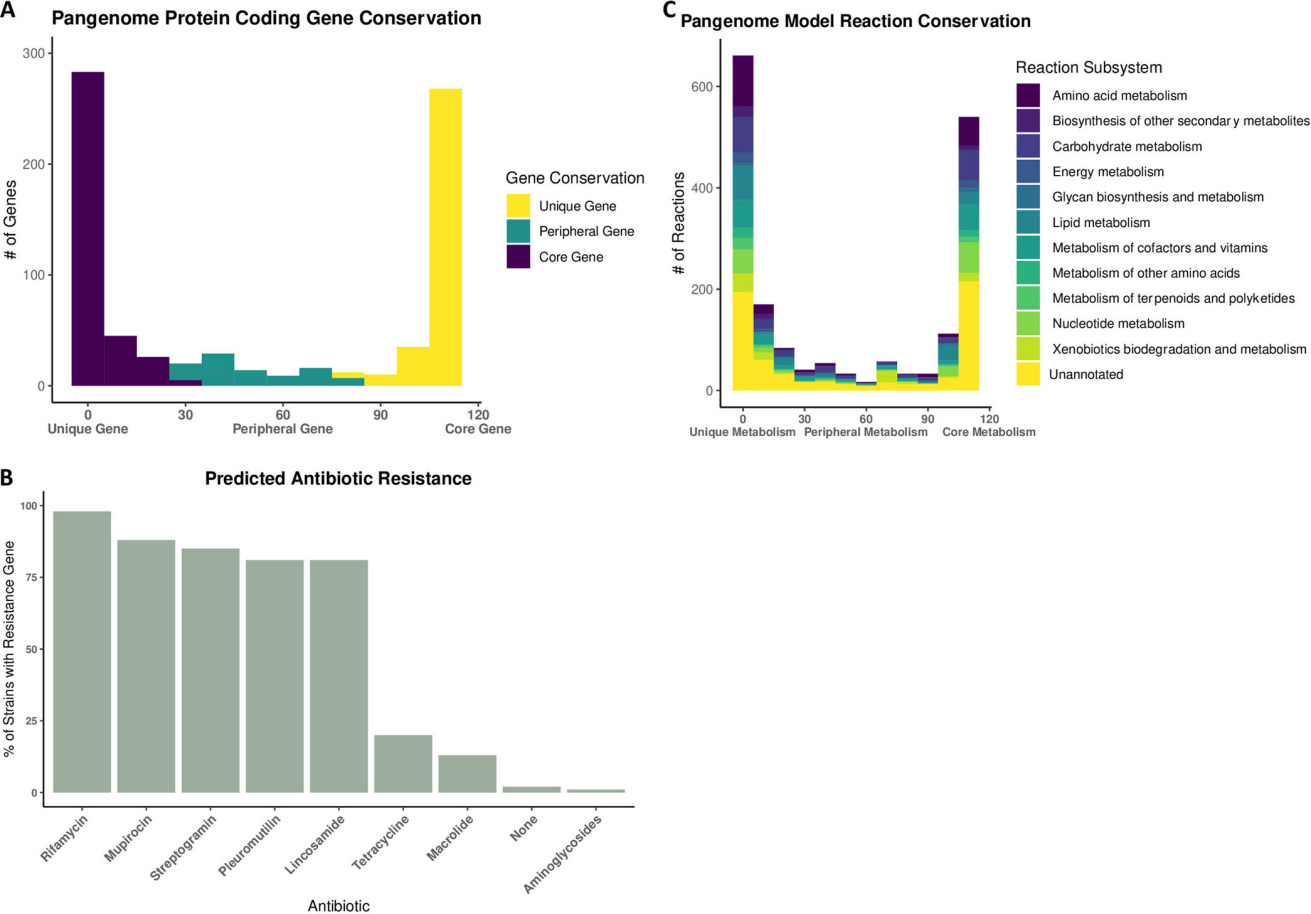
Analysis of the pangenome. (A) Genetic conservation based on protein-encoding gene presence. (B) Predicted prevalence of antibiotic resistance genes based on the Resistance Gene Identifier protein sequence annotation. (C) Pangenome metabolic conservation based on model reaction presence.

Based on the KEGG ortholog identifications of each gene present across the *Gardnerella* pangenome, 359 genes were considered unique, 90 genes were considered peripheral, and 318 genes were considered core (Fig. 3A). There is a high degree of conservation of genes implicated in antibiotic resistance across the pangenome. A total of 98% of the strains have genes implicated in rifamycin resistance, 88% have genes implicated in mupirocin resistance, 85% have genes implicated in streptogramin resistance, 81% have genes implicated in lincosamide resistance, and 81% have genes implicated in pleuromutilin resistance (Fig. 3B). In the minority are strains that have genes associated with tetracycline resistance (20% of the strains), macrolide resistance (13% of the strains), and aminoglycoside resistance (1% of the strains).

Based on the 110 metabolic models constructed to represent the *Gardnerella* pangenome, 919 reactions were considered unique, 221 were peripheral reactions, and 695 were core reactions (Fig. 3C). Of the 209 reactions associated with amino acid metabolism, 122 were unique reactions (58.4%), compared to 70 core reactions (33.5%). Of the 48 reactions associated with terpenoid and polyketide metabolism, 29 were unique reactions (60.4%), and only 14 were core reactions (29.2%). Additionally, of the 106 reactions associated with xenobiotic biodegradation and metabolism, 55 were unique reactions (51.2%), compared to 22 core reactions (20.8%). Finally, the category glycan biosynthesis and metabolism, composed of 18 reactions, was enriched for core reactions (10; 55.6%) compared to unique reactions (6; 33.3%).

### Gene essentiality.

**FIG 4:**
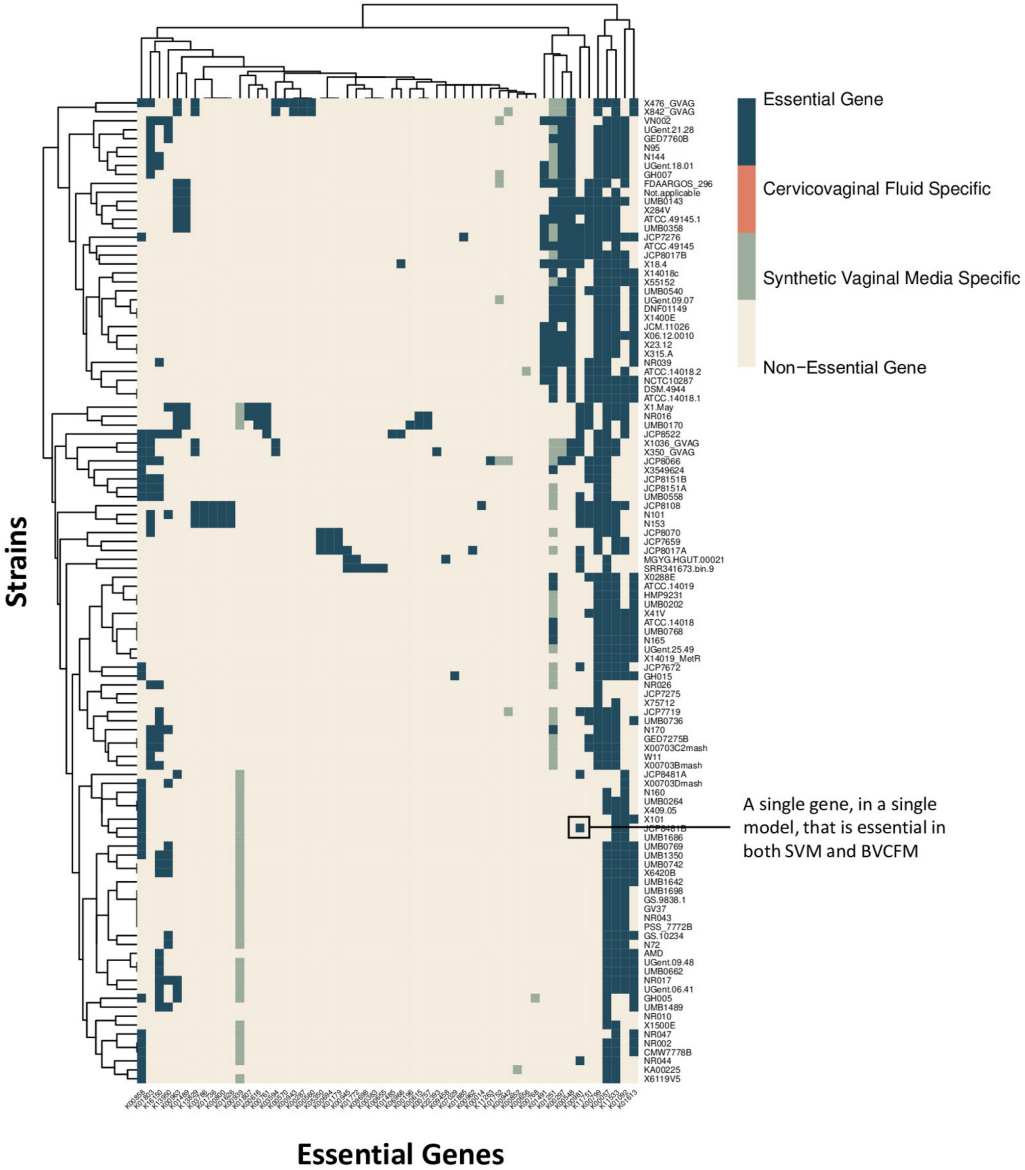
*In silico* gene essentiality screen across 110 models, contextualized to synthetic vaginal medium (SVM) or BV-positive cervicovaginal fluid medium (BVCFM). The text highlighting one block is used to facilitate the interpretation of the data.

Based on the assessed model gene essentiality in both bacterial vaginosis-positive cervicovaginal fluid medium (BVCFM) and synthetic vaginal medium (SVM), 57 genes were identified as being essential across the pangenome (Fig. 4). There is near-universal essentiality of the four following genes: *gpsA* (K00057), *fas* (K11533), *suhB* (K01092), and *psd* (K01613). KEGG annotations indicate that *gpsA* is involved in glycerophospholipid metabolism, *fas* is involved in fatty acid biosynthesis, *suhB* is involved in inositol phosphate metabolism, and *psd* is involved in glycerophospholipid metabolism. Using the DrugBank repository, we identified two potential compounds capable of targeting *fas*-related fatty acid synthesis, pyrazinamide and pretomanid (20). Both drugs are currently approved for the treatment of tuberculosis but have not been investigated for the treatment of *Gardnerella* infection or BV.

### Model flux comparisons.

**FIG 5:**
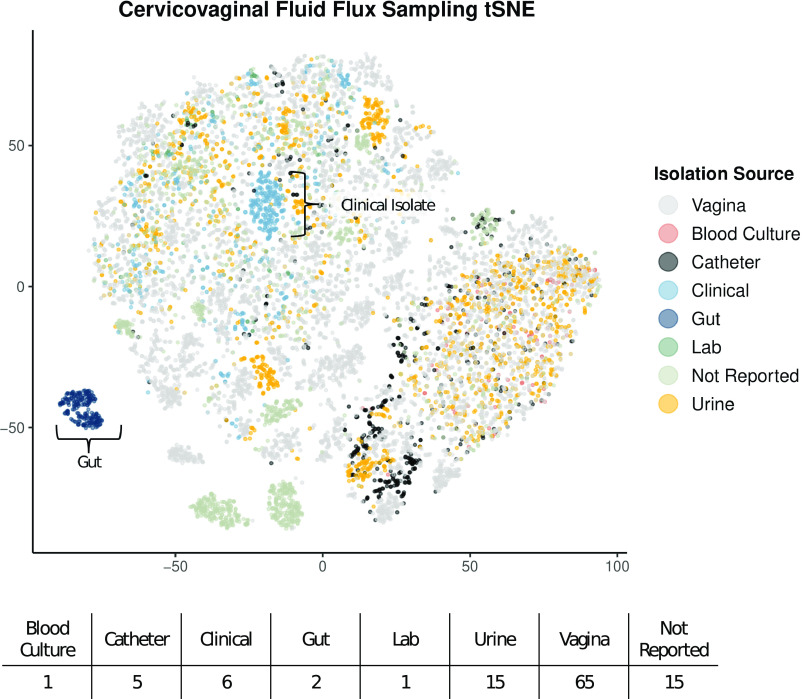
Model flux comparison based on 100 flux samples across all reactions via tSNE dimensionality reduction and the associated number of strains per isolation source.

**FIG 6:**
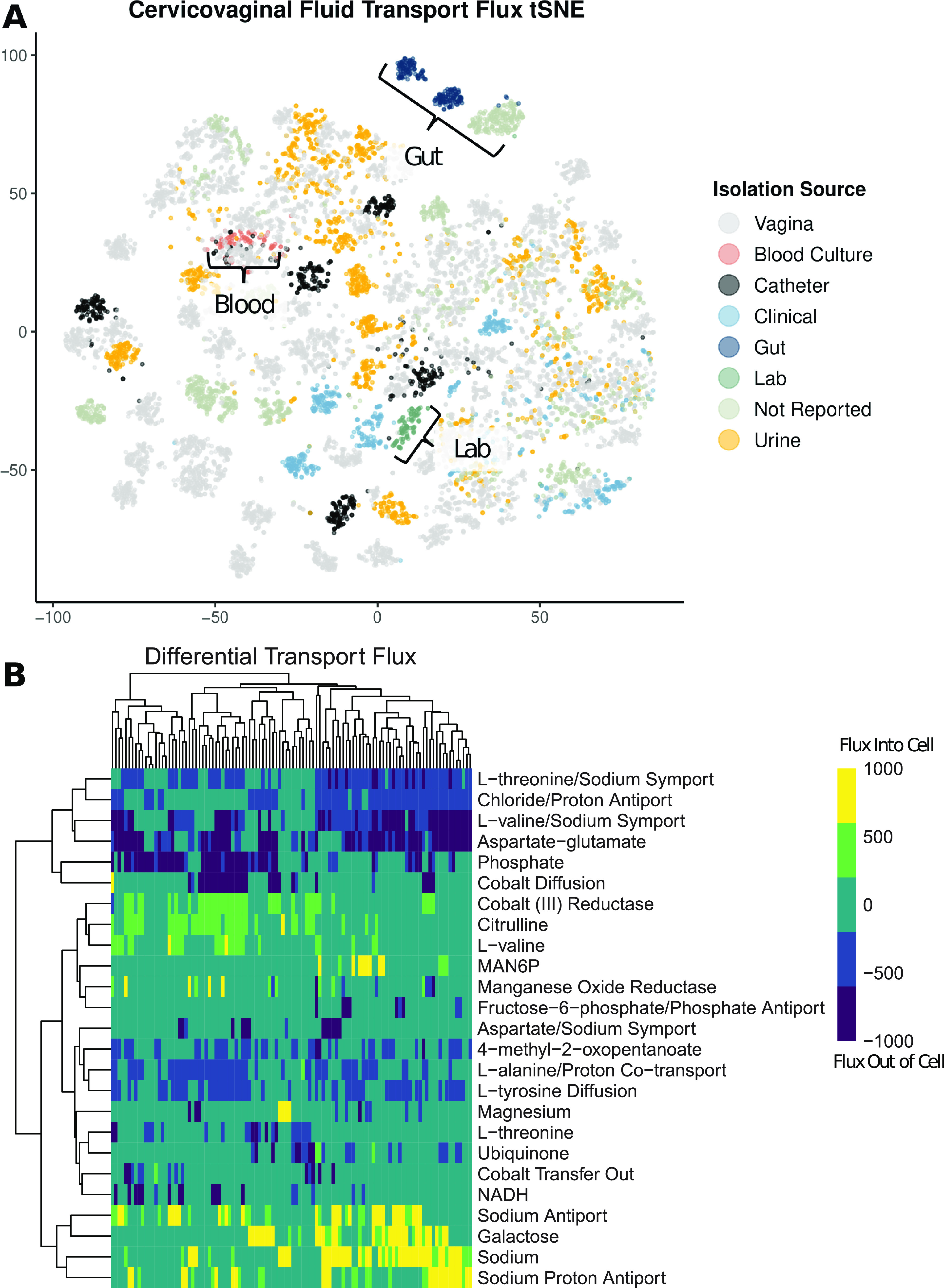
(A) Model flux comparison based on 100 flux samples across transport reactions via tSNE dimensionality reduction. (B) Heat map of median flux values for each model based on the top 25 most varied transport flux reaction values. Values were determined based on 100 flux samples collected via GAPSPLIT.

When comparing the 110 metabolic models based on flux sampling and dimensionality reduction via *t*-distributed stochastic neighbor embedding (tSNE), there is relatively limited clustering based on the sample isolation source (Fig. 5). Samples isolated from the gut and a subset of clinical isolates exhibit clustering. However, when specifically looking at transport flux values alone via tSNE, there is a pronounced clustering of gut isolates, blood culture isolates, and, interestingly, the laboratory 14019_MetR strain (Fig. 6A). A heat map comparison of the transport reactions with the most varied flux values shows that the subset of models that are exporting l-threonine, chloride, l-valine, and aspartate glutamate are also importing galactose and sodium (Fig. 6B). Additionally, a small subset of models are significantly importing mannose-6-phosphate (MAN6P).

## DISCUSSION

The genetic and functional metabolic differences in the *Gardnerella* pangenome remain widely uncharacterized, and an *in vitro* analysis of all publicly available strains would require immense time and resources. This lack of information creates a barrier to the improvement and development of treatments for BV and the subsequent reduction of rates of recurrence. Through *in silico* analysis, we have provided a functional characterization of the *Gardnerella* pangenome. Here, we present the largest set of *Gardnerella* metabolic network reconstructions (GENREs), which characterizes the known *Gardnerella* pangenome. Using these models, we have identified conserved and unique metabolic mechanisms that represent a valuable resource for the future development of therapeutic strategies.

### Pangenome content comparison.

When comparing genetic relatedness to functional relatedness across the *Gardnerella* pangenome, there is a high degree of dissimilarity (61%) between the two dendrograms (Fig. 2B). This result indicates that genetic content similarity does not directly correlate with metabolic functional similarity when investigating the relatedness of *Gardnerella* strains. From an evolutionary perspective, small genetic differences can confer a larger impact on metabolic functionality, as one gene can be involved in multiple reactions. Based on this concept, genetic expression profiles, which may more similarly mirror metabolic functionality (21–23), may offer a more accurate representation of phylogenetic relatedness within the *Gardnerella* pangenome.

With respect to genetic conservation, we specifically investigated the conservation of antibiotic resistance genes due to the translational impact that these genes may have on therapeutic approaches for BV. We identified which drug classes were associated with the most highly conserved antibiotic resistance genes. These drugs included rifamycin, mupirocin, streptogramin, lincosamide, and pleuromutilin (Fig. 3B). Nitroimidazole class antibiotics, the class that metronidazole falls under, are a part of the Resistance Gene Identifier database, but the associated antibiotic resistance genes were not identified within the *Gardnerella* pangenome. Additionally, the large number of antibiotic resistance genes could be a factor contributing to the high rates of BV recurrence, as clindamycin is an antibiotic of the lincosamide class (24).

The high proportion of unique reactions associated with amino acid metabolism (Fig. 3C) is supported by previous literature; differential amino acid metabolism can be used to distinguish *Gardnerella* subgroups (25). Additionally, the large number of unique reactions associated with xenobiotic biodegradation suggests that at the pangenome level, the *Gardnerella* genus is capable of differentially interacting with pharmaceutical treatments as well as nonendogenous probiotics (26). This result speaks to the need for understanding which *Gardnerella* strains are present in BV to adequately and effectively develop patient-specific treatments. Interestingly, the large number of unique reactions associated with terpenoid and polyketide metabolism could offer insight into why some women present with persistent and odorous BV while others remain asymptomatic. Previous research has shown that polyketide metabolism can function as an antimicrobial agent, allowing the polyketide-producing bacteria to outcompete their microbial competitors (27). In short, this result indicates that some *Gardnerella* strains may be uniquely equipped to outcompete others as vaginal microbes. Finally, glycan-related metabolism is uniquely enriched in the pangenome core metabolism. Vaginal mucus is sialoglycan rich and can be utilized by *Gardnerella* as a nutrient if sialidase activity, which hydrolyzes the sialoglycans and frees sialic acid from the glycan chain, is present (28). Interestingly, not all *Gardnerella* strains are sialidase positive (29). Conserved glycan metabolism could indicate the inner pangenome coevolution of *Gardnerella* based on the differences in sialidase activity across strains as well as the potential coevolution with sialic acid-catabolizing microbes such as *Fusobacterium* (30).

### Gene essentiality.

Conserved essential genes could serve as novel targets for drug development. One such essential gene, *gpsA*, is a predicted glycerol-3-phosphate dehydrogenase, and it is involved in phospholipid synthesis. Previous research has identified *gpsA* as a virulence factor in Lyme disease as well as in enhancing nasopharyngeal colonization by Streptococcus pneumoniae (31, 32). Another conserved essential gene, *suhB*, is an inositol monophosphatase. This gene has been shown to regulate multiple virulence factors in Pseudomonas aeruginosa and to play an essential role in Burkholderia cenocepacia biofilm formation, motility, and antibiotic resistance (33, 34). Based on this previous research, both *gpsA* and *suhB* could be universally essential for driving *Gardnerella* virulence as well as *Gardnerella* adaptation to the vaginal mucosal environment. *psd* is a phosphatidylserine decarboxylase that plays a role in bacterial membrane biogenesis and has been identified as a potential antimicrobial target (35). *psd* activity in Plasmodium falciparum has been successfully inhibited using 4-quinolinamine compounds (36). Due to the essentiality of *psd* in *Gardnerella*, 4-quinolinamine compounds may serve as a starting point for novel BV treatment development. Another conserved essential gene, *fas*, is involved in fatty acid biosynthesis, thus playing an essential role in bacterial membrane construction. Because fatty acid synthase type II (FASII) is bacterium specific, *fas* may be a potential novel BV drug target.

### Flux analysis.

By investigating transport-specific flux values, we can make inferences regarding how strains differentially interact with their vaginal metabolic environment. Dimensionality reduction and visualization via tSNE highlight the differential clustering of laboratory strains compared to strains collected from body sites (Fig. 6A). This finding emphasizes the gap in the understanding of *Gardnerella* metabolic functionality due to the primary use of laboratory strains for *in vitro* experimentation. Second, the dispersed nature of vaginal isolates indicates wide variation in the functional metabolism of vaginal microbiome strains and, subsequently, strains involved in BV (Fig. 6B). The differential import of galactose indicates strain-level variation in energy sources (37). There is a small set of strains that have high levels of mannose-6-phosphate import. The mannose-6-phosphate transport reaction was originally characterized in the published Bacillus subtilis 168 metabolic model (38). Bacillus subtilis has been isolated from cervicovaginal fluid and was identified via 16S rRNA gene sequencing (39). Mannose-6-phosphate is an essential ligand for the mannose-6-phosphate enzyme, a key enzyme for lysosomal function (40). Previous research has shown that interrupting mannose-6-phosphate receptor transport from the Golgi apparatus to the endosome reduces lysosomal function and inhibits host lysosome elimination of infection (41, 42). These findings suggest that some strains of *Gardnerella* may sequester mannose-6-phosphate as a mechanism of evading host lysosomal clearance. This phenotype would result in disordered vaginal epithelial cell function due to the lack of waste removal and would likely be concordant with increased inflammation and oxidative stress (43, 44).

### Conclusion.

*Gardnerella* is one of the most abundant genera present in BV. Despite the high prevalence of BV and the associated negative health impacts, the *Gardnerella* pangenome is largely uncharacterized at both the genetic and metabolic function levels. Using *in silico* analysis via genome-scale metabolic models and vaginal metabolic environment contextualization, we studied 110 *Gardnerella* strains. Through these analyses, we discovered that genetic relatedness does not necessarily translate to functional relatedness among *Gardnerella* strains. These findings highlight that BV research should not be overly dependent on the genetic relatedness of strains and instead should focus on the functional understanding of the *Gardnerella* pangenome in order to design effective interventions at a strain-specific level. Conserved gene essentiality predictions, specifically for *gpsA*, *fas*, *suhB*, and *psd*, could inform the development of novel drugs that target this large, diverse genus. While these genes are found in other organisms such as Escherichia coli, there is limited information on compounds that target any of these four genes. Finally, *Gardnerella* strains interact differently with their vaginal metabolic environment, suggesting the potential for metabolic niche development within the pangenome. These discoveries serve as a starting point for developing a deeper understanding of patient-level variation in BV and its impact on health outcomes and infection and for translating these findings to develop personalized therapeutic approaches.

## MATERIALS AND METHODS

### Model construction and contextualization.

To perform metabolic characterization of the *Gardnerella* pangenome *in silico*, we identified 110 *Gardnerella* whole-genome sequences considered to be of “good” quality from the Bacterial and Viral Bioinformatics Resource Center (BV-BRC) 3.6.12 database (45). BV-BRC guidelines define good as “a genome that is sufficiently complete (80%), with sufficiently low contamination (10%),” and amino acid sequences that are at least 87% consistent with known protein sequences. The corresponding amino acid sequences of these 110 strains were then annotated via RAST 2.0 (46–48). The Reconstructor algorithm was used to create GENREs for each strain (49). Model quality was assessed using the field-standard MEMOTE score (50); the results are included in the associated GitHub repository (https://github.com/emmamglass/Gardnerella_Pangenome). After network construction, two *in silico* medium conditions were defined, synthetic vaginal medium (SVM) and bacterial vaginosis-positive cervicovaginal fluid medium (BVCFM). The SVM condition was based on the composition of previously defined *in vitro* media specific for the growth of vaginal microflora, on which *Gardnerella* has been shown to successfully grow *in vitro* (51). The BVCFM condition was based on previous metabolomics analysis of cervicovaginal fluid collected from both healthy and BV-positive patients (52). The *in silico* BVCFM was enriched for metabolites that had significantly higher levels in BV-positive cervicovaginal fluid samples in order to specifically investigate metabolic functionality in the disease state. Transport reactions absent from initial model construction but required for *in silico* medium metabolite usage were added as needed to the reconstructions in addition to the respective exchange reactions (Fig. 1).

### Model comparisons.

Gene presence for each model was initially determined by the BLASTp output annotations (53). For each BLASTp protein-encoding gene, the associated KEGG ortholog was identified and used to construct a binary matrix based on the presence or absence of the gene in each model. In parallel, a model reaction presence binary matrix was also constructed. Each binary matrix was constructed based on the pangenome of reactions/genes present across all 110 strain network reconstructions. Each binary matrix indicates at the strain level whether a reaction or gene is present (1) or absent (0) for each network reconstruction. The respective dendrograms of gene presence and reaction presence were constructed using the dendextend package in R (54). Dissimilarity matrices were constructed using the Sørensen-Dice method (55). Hierarchical clustering was performed using the Ward method (56). Entanglement values of the two dendrograms were calculated using the entanglement function in dendextend. Four k-means clusters are represented by branch coloring for each of the respective dendrograms. For assessing protein-encoding gene conservation across models, the binary matrix of gene presence was used to determine how many models a gene was or was not present in. Genes present in >75% of the models were considered core genes, genes present in 25 to 75% of the models were considered peripheral genes, and genes present in <25% of the models were considered unique genes. An analogous metric was used to define model reaction conservation. Reaction subsystems were determined based on the corresponding KEGG reaction metabolic subsystem annotations. Predicted antibiotic resistance was determined with the Resistance Gene Identifier 5.2.1 platform using the amino acid sequence of each strain (57). Genes were considered a match if there was a >80% regional match based on protein sequence, indicating a conserved mutant allele.

### Gene essentiality.

The corresponding exchange reactions were opened (flux bounds of −1,000 to 1,000) for the associated components of the SVM and BVCFM to create two contextualized models for each of the 110 strains (see Table S1 in the supplemental material for *in silico* medium configurations). Gene essentiality was determined using the gene essentiality function in the COBRApy toolbox (58). This function simulates single-gene deletions for every gene present in a model. If a deletion results in a >80% reduction in flux through the objective function (biomass synthesis) as determined from the simulation of the wild-type condition in the respective media of interest, the gene is categorized as being essential. The KEGG ortholog values for each essential gene were determined and used for further analysis. After running the gene essentiality screen, a heat map was generated using the pheatmap package in R (59). A Euclidean distance matrix was constructed and then clustered using the complete clustering method.

10.1128/msystems.00689-22.1 TABLE S1 *In silico* medium components, including the respective flux bounds and modelSEED identifiers. Download Table S1, XLSX file, 0.01 MB. Copyright © 2022 Dillard et al. 2022 Dillard et al. https://creativecommons.org/licenses/by/4.0/ This content is distributed under the terms of the Creative Commons Attribution 4.0 International license.

### Flux comparison.

Using the COBRApy-compatible GAPSPLIT function (60), 100 flux samples were collected for each model in the cervicovaginal medium context (60). To better understand the inherent clustering of strains based on simulated flux distributions, we utilized dimensionality reduction and visualization via tSNE on the collected flux samples. tSNE analysis was run via the sci-kit learn sklearn.manifold package in Python using default parameters (61, 62). Strain metadata from BV-BRC were used to map sample isolation sources for tSNE visualization. Additionally, transport reactions were isolated from the flux sampling data, and the median values for each model’s transport reaction were used to create a heat map comparing the top 25 most variable transport reaction fluxes across the models. The pheatmap library in R was used for heat map construction, which uses Euclidean distance and complete clustering to determine the hierarchical structure.
